# Quantitative proteomics reveals the effect of Yigu decoction (YGD) on protein expression in bone tissue

**DOI:** 10.1186/s12014-021-09330-0

**Published:** 2021-10-12

**Authors:** Ruikun Zhang, Kun Yan, Yulun Wu, Xinmiao Yao, Guijin Li, Linpu Ge, Zhineng Chen

**Affiliations:** 1 The Third Clinical Medical College of Zhejiang, Chinese Medical University, Zhejiang, Hangzhou, 310053 China; 2 Department of Orthopedics, The Third Affiliated Hospital of Zhejiang, Chinese Medical University, Zhejiang, Hangzhou, 310005 China; 3 Rehabilitation Medicine Center of Zhejiang Provincial People’s Hospital, Rehabilitation & Sports Medicine Research Institute of Zhejiang Province, People’s Hospital of Hangzhou Medical College, Hangzhou, 310014 China

**Keywords:** Yigu decoction, Osteoporosis, TMT proteomics, Bone metabolism

## Abstract

**Background:**

Osteoporosis (OP) is a systemic bone disease characterized by decreased bone mass, destruction of the bone tissue microstructure, increased bone brittleness and an increased risk of fracture. OP has a high incidence rate and long disease course and is associated with serious complications. Yigu decoction (YGD) is a compound prescription in traditional Chinese medicine that is used to treat OP. However, its mechanism in OP is not clear. This study used a tandem mass tag (TMT)quantitative proteomics method to explore the potential bone-protective mechanism of YGD in an osteoporotic rat model.

**Materials and methods:**

A rat model of OP was established by ovariectomy. Eighteen 12-week-old specific-pathogen-free female Wistar rats weighing 220 ± 10 g were selected. The eighteen rats were randomly divided into 3 groups (n = 6 in each group): the normal, model and YGD groups. The right femurs from each group were subjected to quantitative biological analysis. TMT quantitative proteomics was used to analyze the proteins extracted from the bone tissue of rats in the model and YGD groups, and the differentially expressed proteins after intervention with YGD were identified as biologically relevant proteins of interest. Functional annotation correlation analysis was also performed to explore the biological function and mechanism of YGD.

**Result:**

Compared with the model group, the YGD group showed significant upregulation of 26 proteins (FC > 1.2, P < 0.05) and significant downregulation of 39 proteins (FC < 0.833, P < 0.05). Four important targets involved in OP and 5 important signaling pathways involved in bone metabolism were identified.

**Conclusions:**

YGD can significantly increase the bone mineral density (BMD) of osteoporotic rats and may play a therapeutic role by regulating target proteins involved in multiple signaling pathways. Therefore, these results improve the understanding of the OP mechanism and provide an experimental basis for the clinical application of YGD in OP treatment.

**Supplementary Information:**

The online version contains supplementary material available at 10.1186/s12014-021-09330-0.

## Background

Osteoporosis (OP) is a bone disease characterized by low bone mass, bone tissue destruction and pain [1]. The main manifestations are low bone mass, bone tissue metamorphism, destruction of the bone structure, reduced bone strength and an increased risk of fracture [2]. Low bone mass is characterized by decreased bone mineral density (BMD) and increased bone brittleness. Bone tissue metamorphism and bone structure destruction are bone homeostasis changes caused by decreased osteoblast production and increased osteoclast proliferation, and fracture, mainly vertebral fracture, hip fracture or forearm fracture, is the end point event of OP. Fracture caused by OP increases the medical burden [3, 4], and refracture [5–7] and death after fracture are also harmful to public health, which necessitates studies on the etiology of OP. Some studies have attempted to explain the pathogenesis of OP via different basic research methods and identify therapeutic targets by proteomic and metabonomic investigation of the pathogenic mechanism of OP. Previous studies [8–12] have shown that a variety of signaling pathways, such as the ER pathway, OPG/RANKL pathway, BMP-2/Smad pathway, classical Wnt/β-catenin signaling pathway, and PI3K/akt signaling pathway, can affect osteoblast proliferation and inhibit osteoclast differentiation.

During the development of modern medicine, due to the influence of cultural exchange and integration, traditional Chinese medicine (TCM) has attracted increasing attention because of its unique role. Researchers have extracted active ingredients from various herbs to treat different diseases. Yigu decoction (YGD) consists of *Fructus psoraleae, Rhizoma Drynariae, Radix Rehmanniae, Salvia miltiorrhiza, Epimedii Folium* and *Dioscorea opposita*. *Fructus psoraleae* could reportedly improve the damage caused by bone resorption at the level of bone metabolism [13] and increase BMD in mice [14]. Studies have confirmed that *Rhizoma Drynariae* has antioxidant effects on osteoblasts [15]. The use of Radix *Rehmannia* extract increased the thickness of cortical bone and the number of trabeculae in the bone marrow cavity of osteoporotic rats [16]. According to previous studies, the extract of *Salvia miltiorrhiza* could improve BMD [17] and increase the levels of alkaline phosphatase and tartrate resistant acid phosphatase (TRACP) [18]. The extract of *Epimedii Folium* can significantly increase the expression of transforming growth factor-beta (TGF-β), reduce the movement and bone resorption activity of isolated osteoclasts [19], accelerate bone formation, and contribute to bone plastic surgery and remodeling [20]. A literature search revealed that *Dioscorea opposita* is beneficial for the proliferation of osteoblasts and inhibits the differentiation of osteoclasts [21]. In basic research, our team found that YGD could increase the proliferation and differentiation of osteoblasts [22, 23], increase the serum levels of bone Gla protein (BGP) and Ca^2+^ [24], improve the biomechanical properties of bone [25, 26], and promote the expression of osteoblast BMP [25, 27] to ameliorate the inhibitory effect on bone resorption and achieve the prevention and treatment of OP. The effects of YGD in the treatment of OP are mainly achieved by promoting osteoblast proliferation. Our previous research [26–30] mainly focused on the effect of YGD on the classical Wnt/β-catenin and BMP-2/Smad signaling pathways in the bone tissue of osteoporotic rats. However, whether YGD is also involved in the regulation of other proteins and the expression of signaling pathway components warrants further study. Microarray studies have been performed to explore the pathogenesis of OP. A larger-sample size expression profile can be obtained by integrated microarray analysis [37], which is helpful for identifying the differentially expressed genes (DEGs) in OP more accurately than a single microarray. Based on the above research, we established an OP model in ovariectomized rats and treated them with YGD. The use of TMT quantitative proteomics technology to study protein expression differences, mass spectrometry experiments and data analysis led to possible conclusions, which guided subsequent research.

## Materials and methods

### Drugs and reagents

YGD contains six kinds of traditional Chinese medicine: *Rhizoma Drynariae, Radix Rehmanniae, Fructus psoraleae, Salvia miltiorrhiza, Epimedii Folium, and Dioscorea opposita,* which were purchased from *The Third Affiliated Hospital of Zhejiang Chinese Medical University.*

### Animal culture

Eighteen12-week-old specific-pathogen-free female Wistar rats weighing 220 ± 10 g were used in this study. All the experimental animals were provided by Shanghai Shrek Experimental Animal Co., Ltd. (license number: SCXK, Shanghai 2013–0016, certificate code: 2013001806499). The rats were raised in the Animal Experimental Center of Zhejiang University of Traditional Chinese Medicine (laboratory facility license number: SYXK [Zhejiang] 2013–0184). The feeding room was well ventilated, the room temperature was controlled at 21 ± 1 °C, the humidity was 63%, the noise was less than 55 decibels, food and water were provided ad libitum, and light/dark conditions were alternated every 12 h. All animals received meticulous and humanitarian care.

Eighteen rats were randomly divided into 3 groups (6 rats in each group): the normal group, model group and YGD group. Under anesthesia via an intraperitoneal injection of ketamine (5 mg 100 g^−1^), rats in the model group and YGD group were ovariectomized. Three days after the operation, each rat was injected with 40,000 U/d penicillin intramuscularly to prevent infection. The rats in the normal group did not receive any treatment. There was no death of rats after the operation, and the success rate of the model was 100%. After the operation, the rats were fed a routine diet for 12 weeks, and the drug was given once a day from the 13th week. The rats in the normal group and the model group were perfused with 10 ml·kg^−1^ double distilled water, and the rats in the YGD group were fed 10 ml kg^−1^ water extract of YGD. The whole process lasted for 12 weeks.

After 12 weeks, the rats were anesthetized via injection of 10% pentobarbital sodium in the right femur, and the left ventricle was intubated and perfused with normal saline. All bone tissues were quickly frozen and preserved at − 80 °C.

### Determination of BMD in rats

A dual-energy X-ray bone densitometer was used to measure the BMD of the small animals (produced by GE, USA, Lunar Prodigy). The femur was placed on the measuring table for automatic measurement.

### HE staining

The bone tissue of the rats was removed, fixed with 4% paraformaldehyde (in 0.1 MPBS containing 0.1% DEPC, pH 7.0–7.6), and decalcified with 10% EDTA. The distal femoral intercondylar fossa was cut lengthwise into a 1.5 cm bone segment. Tissue samples were processed by conventional paraffin embedding, sectioning, processing, and HE staining and examined by light microscopy.

### Protein extraction and peptide enzymatic hydrolysis

The rats were anesthetized with 10% pentobarbital sodium at 0.3 ml 100 g^−1^. The blood was collected from the heart in vitro, placed at room temperature for 40 min at 4 °C, and centrifuged at 3600 rpm for 15 min. The upper yellowish serum was transferred into a new centrifuge tube. The sample was filtered through a 0.22 µm membrane, sterilized on an ultraclean worktable and then placed in a freezer at -80 °C for long-term storage.

SDT (4% SDS, 100 mM Tris–HCl, pH 7.6, 0.1 M DTT) buffer was used for sample lysis and protein extraction. The amount of protein was quantified with the BCA Protein Assay Kit (Bio-Rad, USA). Protein digestion by trypsin was performed according to the filter-aided sample preparation (FASP) procedure described by Matthias Mann. The digested peptides of each sample were desalted on C18 Cartridges (Empore SPE Cartridges C18 [standard density], bed I.D. 7 mm, volume 3 ml, Sigma), concentrated by vacuum centrifugation and reconstituted in 40 µl of 0.1% (v/v) formic acid (FA).

## TMT labeling

For each sample, a 100 μg of peptide mixture was labeled using TMT reagent according to the manufacturer’s instructions (Thermo Scientific). The tagging information is shown in the table below. There were 3 groups designed for the project, and each group contained 3 or 2 biological repetitive samples, for a total of 8 samples (Table 1).

**Table 1:** TMT labeling scheme for the 8 samples

TMT labelling	126	127_N	127_C	128_N	128_C	129_N	129_C	130_N	No
Sample name	Control-1	Control-2	Control-3	Model-1	Model-2	Model-3	YGD-1	YGD-2	1

No. experimental group (PLEX) number was marked

### Reversed-phase (RP) grading

The labeled peptides in each group were mixed in equal amounts and graded by a High pH Reversed-Phase Peptide Fractionation Kit. First, acetonitrile and 0.1% trifluoroacetic acid (TFA) were used for column equilibration, and then, the mixed labeled peptide samples were added to the column with pure water and desalted at low speed by centrifugation. Finally, the column-bound peptides were gradient eluted with high-pH acetonitrile solutions of progressively increasing concentrations. After vacuum drying, 12 μl of 0.1% FA was used to dissolve the freeze-dried sample, and the peptide concentration was determined by measuring the OD280.

### LC–MS/MS data acquisition

LC–MS/MS analysis was performed on a Q Exactive mass spectrometer (Thermo Scientific) that was coupled to an Easy nLC (Proxeon Biosystems, now Thermo Fisher Scientific) for 90 min. The peptides were loaded onto a reversed-phase trap column (Thermo Scientific Acclaim PepMap100, 100 μm * 2 cm, nanoViper C18) connected to a C18 reversed-phase analytical column (Thermo Scientific Easy Column, 10 cm long, 75 μm inner diameter, 3 μm resin) in buffer A (0.1% FA) and separated with a linear gradient of buffer B (84% acetonitrile and 0.1% FA) at a flow rate of 300 nl/min controlled by IntelliFlow technology. The mass spectrometer was operated in positive ion mode. MS data were acquired using a data-dependent top10 method by dynamically choosing the most abundant precursor ions from the survey scan (300–1800 m/z) for HCD fragmentation. The automatic gain control (AGC) target was set to 3e6, and the maximum injection time was set to 10 ms. The dynamic exclusion duration was 40.0 s. Survey scans were acquired at a resolution of 70,000 at 200 m/z, the resolution for the HCD spectra was set to 17,500 at 200 m/z, and the isolation width was 2 m/z. The normalized collision energy was 30 eV, and the underfill ratio, which specifies the minimum percentage of the target value likely to be reached at the maximum fill time, was defined as 0.1%. The instrument was run with peptide recognition mode enabled.

### Data analysis

The MS raw data for each sample were searched using the MASCOT engine (Matrix Science, London, UK; version 2.2) embedded into Proteome Discoverer 1.4 for identification and quantitation analysis.

### Bioinformatics analysis

Cluster 3.0 (http://bonsai.hgc.jp/~mdehoon/software/cluster/software.htm) and Java Treeview (http://jtreeview.sourceforge.net) were used to perform hierarchical clustering analysis; the Euclidean distance algorithm was selected for similarity measurement and the average linkage clustering algorithm for clustering. A heat map is often presented as a visual aid in addition to the dendrogram.

Blast2GO (https://www.blast2go.com/) was used to annotate the target protein set with GO, and the process can be summarized into four steps: sequence alignment (Blast), GO entry extraction (mapping), GO annotation (annotation) and InterProScan supplementary annotation (annotation augmentation).

The Kyoto Encyclopedia of Genes and Genomes (KEGG) pathway of the target protein set was annotated by KAAS (KEGG Automatic Annotation Server) software. All differentially expressed proteins were compared to all of the experimentally identified proteins with KEGG annotation results to reveal the enriched pathways as determined by Fisher's exact test.

Based on the PPI relationship in the STRING database, a PPI network map was constructed for the differentially expressed proteins in the comparison group by using CytoScape software. The direct and indirect interactions between the target proteins were found based on the information in the STRING (http://string-db.org/) database, and the interaction network was generated and analyzed by using CytoScape software (version number: 3.2.1).

### Statistical analysis

All the data were from at least three independent experiments and are expressed as the mean ± standard deviation (x ± s). The univariate comparison between the two groups was analyzed by *Student's* T-test in GraphPad Prism software, and the multivariate data were compared by analysis of variance. The difference was statistically significant (P < 0.05).

## Results

### The ability of YGD to alleviate OP

**Fig. 1:**
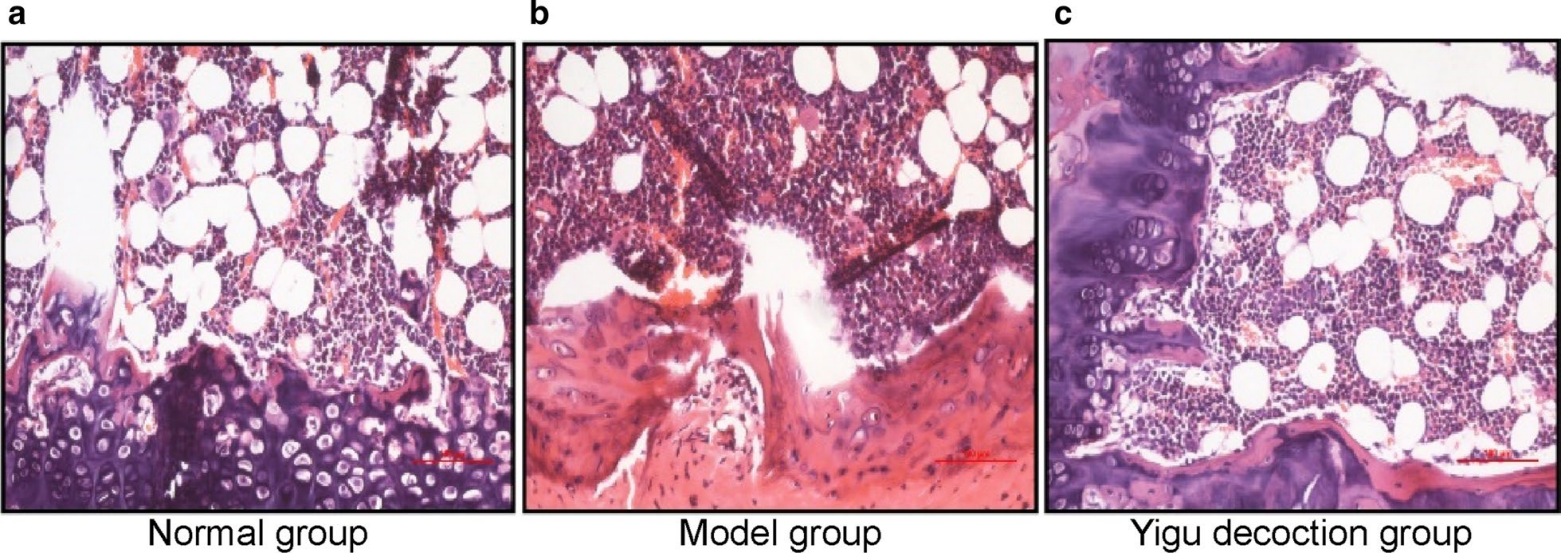
Pathological observation of the right femur of rats (HE staining, 100×)

To explore the ability of YGD to alleviate OP, we first constructed a rat model of OP and measured bone density. The results showed that the bone density value of the model group was significantly lower than that of the normal group (P < 0.01), which indicated that the OP rat model was successfully established. Compared with that of the model group, the BMD of the YGD group increased significantly, and the difference was statistically significant (P < 0.01) (Table 2). In parallel, our team observed the pathological histomorphology of the left femur of the rats. The visible area of the trabecular bone was smaller, the width was narrower, the cortex was thinner, the number was reduced, the morphology and structure were irregular, the arrangement was disordered, and the marrow was expanded in the model group compared with the normal group. After YGD administration for 3 months, the rats in the YGD group were similar to those in the blank group. The bone trabecula was slightly thinner, the shape remained regular, the arrangement remained orderly, and the gap was slightly enlarged in some areas (Fig. 1).

**Table 2:** Comparison of bone mineral density results of rats in each group (x ± s) (unit: g/cm^2^)

Group	Quantity	Femur bone density (g/cm^2)
Normal	6	0.247 ± 0.020
Model	6	0.169 ± 0.013^▲▲^
YGD	6	0.236 ± 0.013^▼▼^

^▲▲^ P < 0.01 compared with the normal group, ^▼▼^ P < 0.01 compared with the model group

### TMT quantitative proteomic analysis of differentially expressed proteins after YGD treatment

To further explore the mechanism of action of YGD in the treatment of OP, through TMT quantitative proteomics analysis, a total of 340,987 secondary spectra were obtained from bone tissues from the control group (control), OP model group (model) and OP model intervention group (YGD). The total number of spectra with matches in the database was 29,695, including 3382 proteins, and 3363 matches showed high similarity (Additional file 1, Fig. 2).

**Fig. 2:**
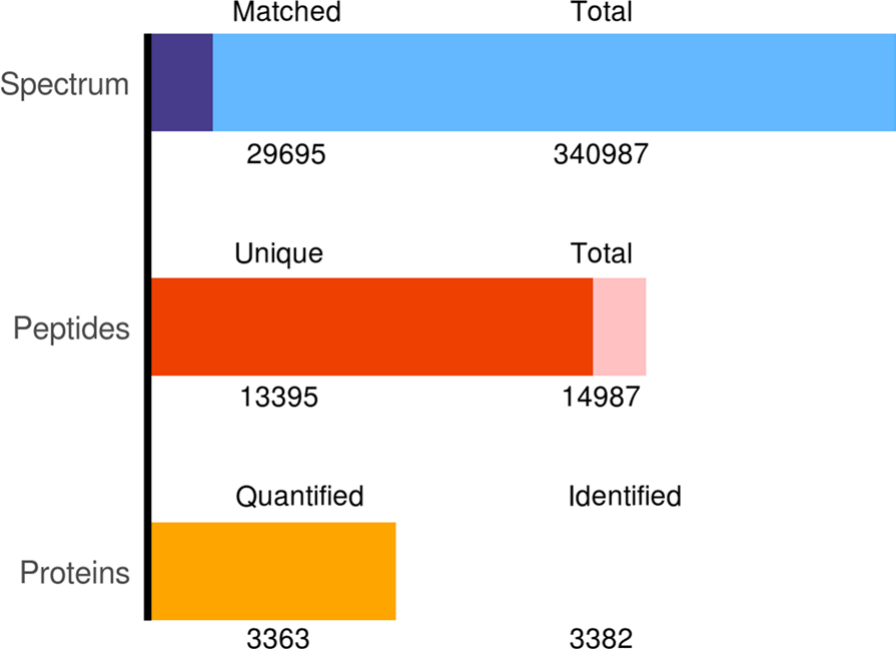
Statistical picture of identification and quantitative results

### Bioinformatics analysis of differentially expressed proteins after YGD treatment

**Fig. 3:**
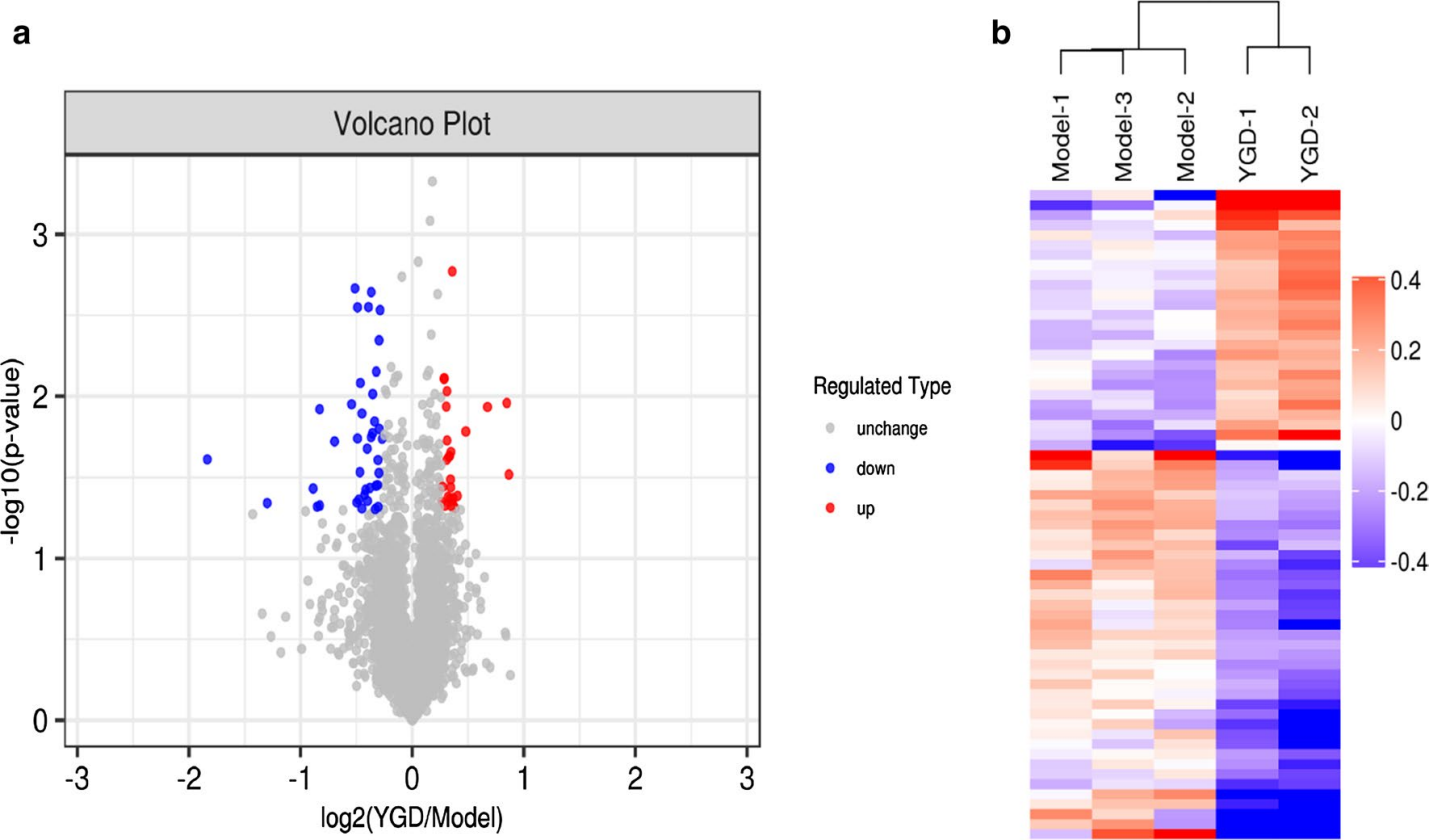
TMT quantitative proteomic analysis of differentially expressed proteins after YGD treatment. **a** The volcano plot was drawn using two factors, log2 fold change (log2FC) and *P* value (Logarithmic transformation based on 10, *Student*'s t-test). The significantly downregulated proteins are annotated in blue (FC < 0.833 and P < 0.05), the significantly upregulated proteins are annotated in red (FC > 1.2 and P < 0.05), and the proteins without differences are indicated in gray. **b** The hierarchical clustering results are presented as a tree-type heatmap with the abscissa showing the sample information and the ordinate showing the significantly differentially expressed proteins, and the expression amounts of the significantly different proteins in different samples were exhibited in the Heatmap by different colors after normalization using the log2 method. Where red represents the significantly upregulated proteins, blue represents the significantly downregulated proteins and grey represents proteins with no quantitative information

To analyze the differentially expressed proteins between different groups, the experimental data were further screened. Biological analysis of the differentially expressed proteins showed that a total of 65 proteins were significantly affected after treatment. Compared with the model group, the YGD group showed significant upregulation of 26 proteins and downregulation of 39 proteins (FC ≥ 1.2, P < 0.05). To determine whether the differential protein expression changes represent significant effects caused by biological treatment of the samples, a hierarchical clustering algorithm was used to group the differentially expressed proteins of the compared groups and present them as a heat map. The heat map of the results shows that the significantly differentially expressed proteins can effectively separate the comparison groups, indicating that the screening of differentially expressed proteins can represent the impact of biological treatment on the samples. The detailed analysis is shown in Fig. 3. (Please refer to Additional file 2 for the specific proteins.)

### Functional analysis of differentially expressed proteins

**Fig. 4:**
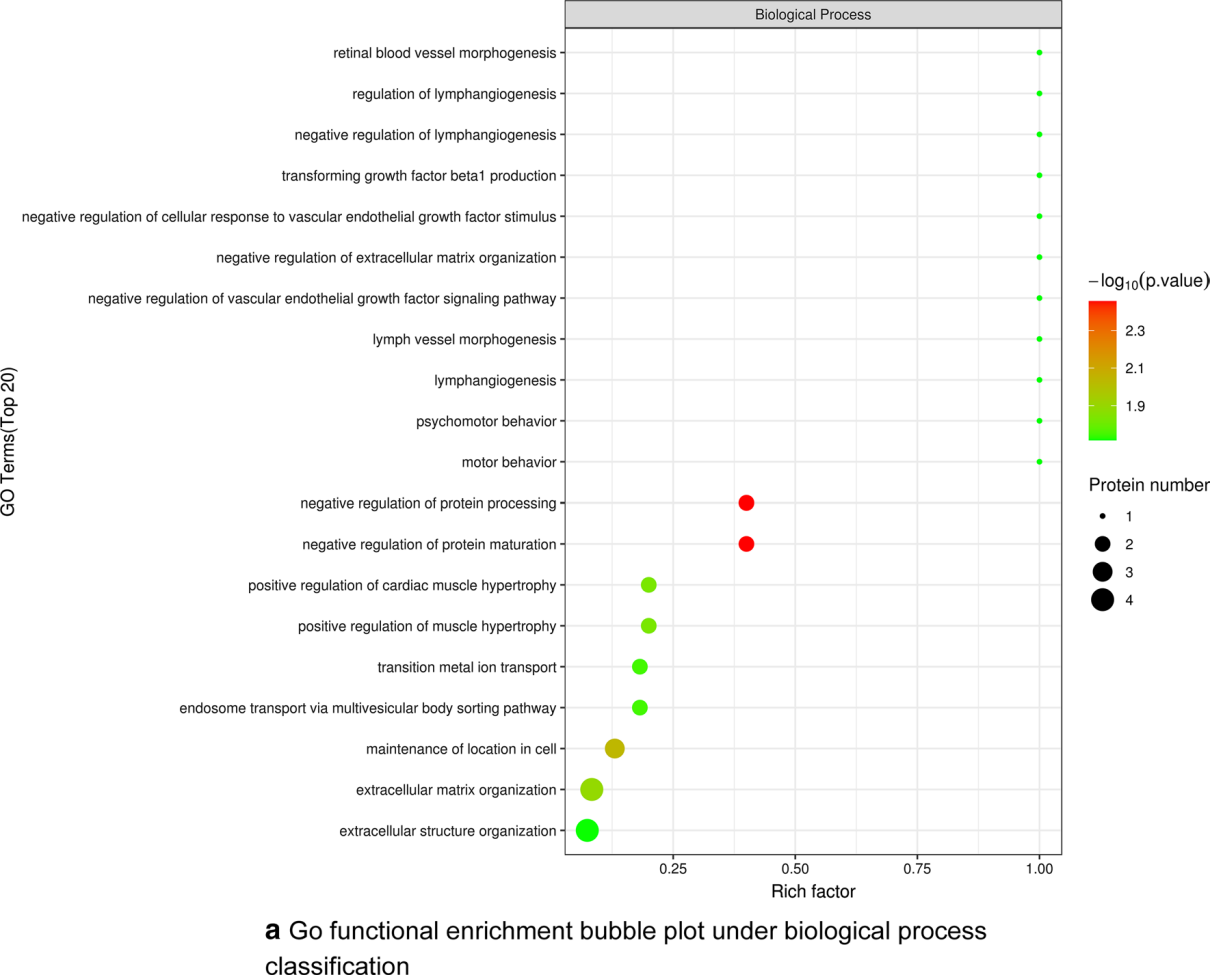

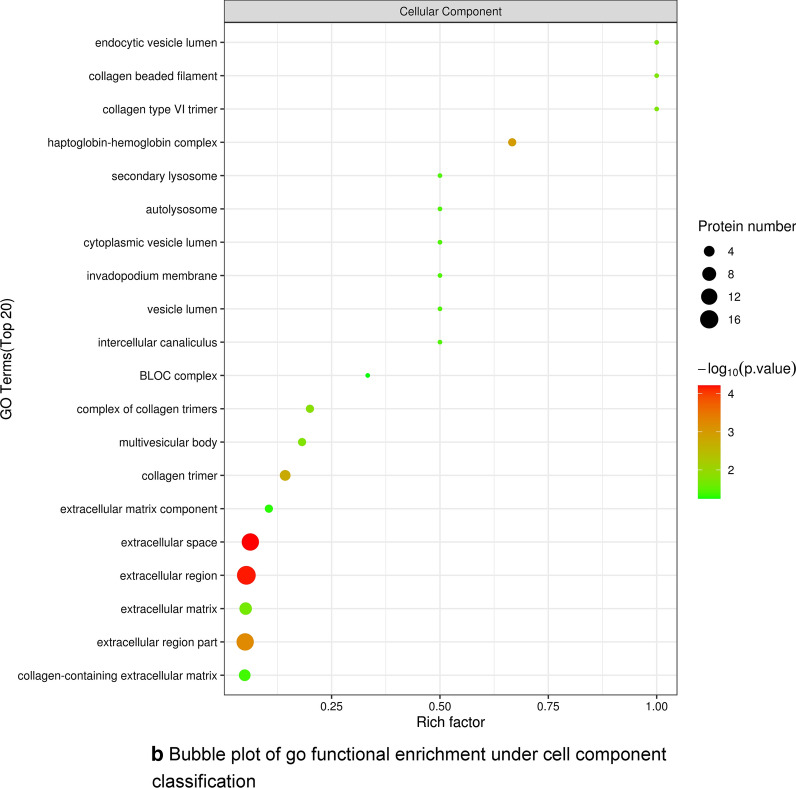

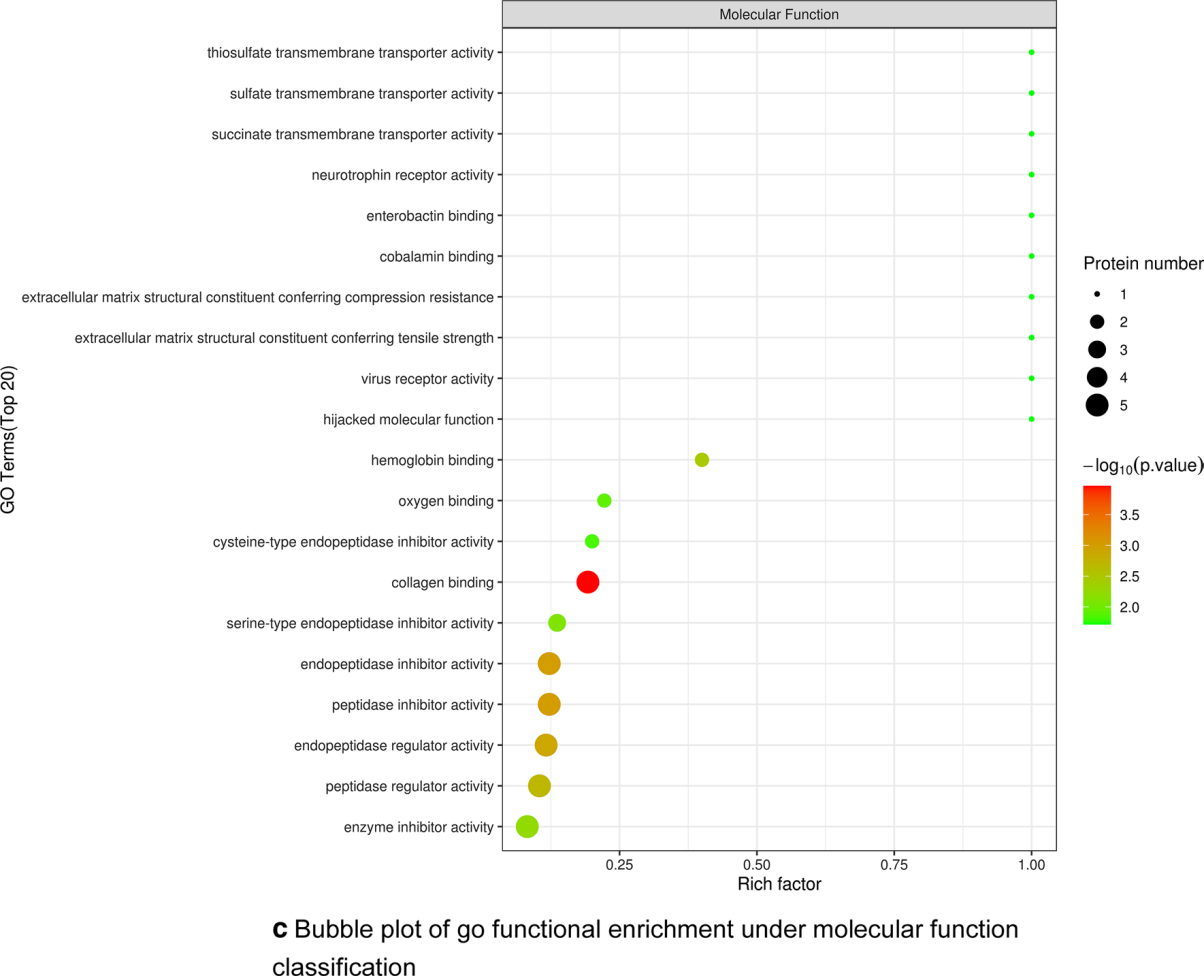
**a** Go functional enrichment bubble plot under biological process classification. **b** Bubble plot of go functional enrichment under cell component classification. **c** Bubble plot of go functional enrichment under molecular function classification

To better understand the biological functions of differentially expressed proteins after YGD treatment, we carried out functional annotation via GO and KEGG analysis. A total of 1656 GO terms were obtained by GO functional enrichment analysis, including 1195 biological process (BP) entries, 218 cellular component (CC) entries and 243 molecular function (MF) entries. The first 20 items are shown in Fig. 4a–c). Significant differences are observed in biological processes related to OP, such as protein regulation and muscle regulation, the molecular function of enzyme regulation, and the localization protein collagen trimer.

**Fig. 5:**
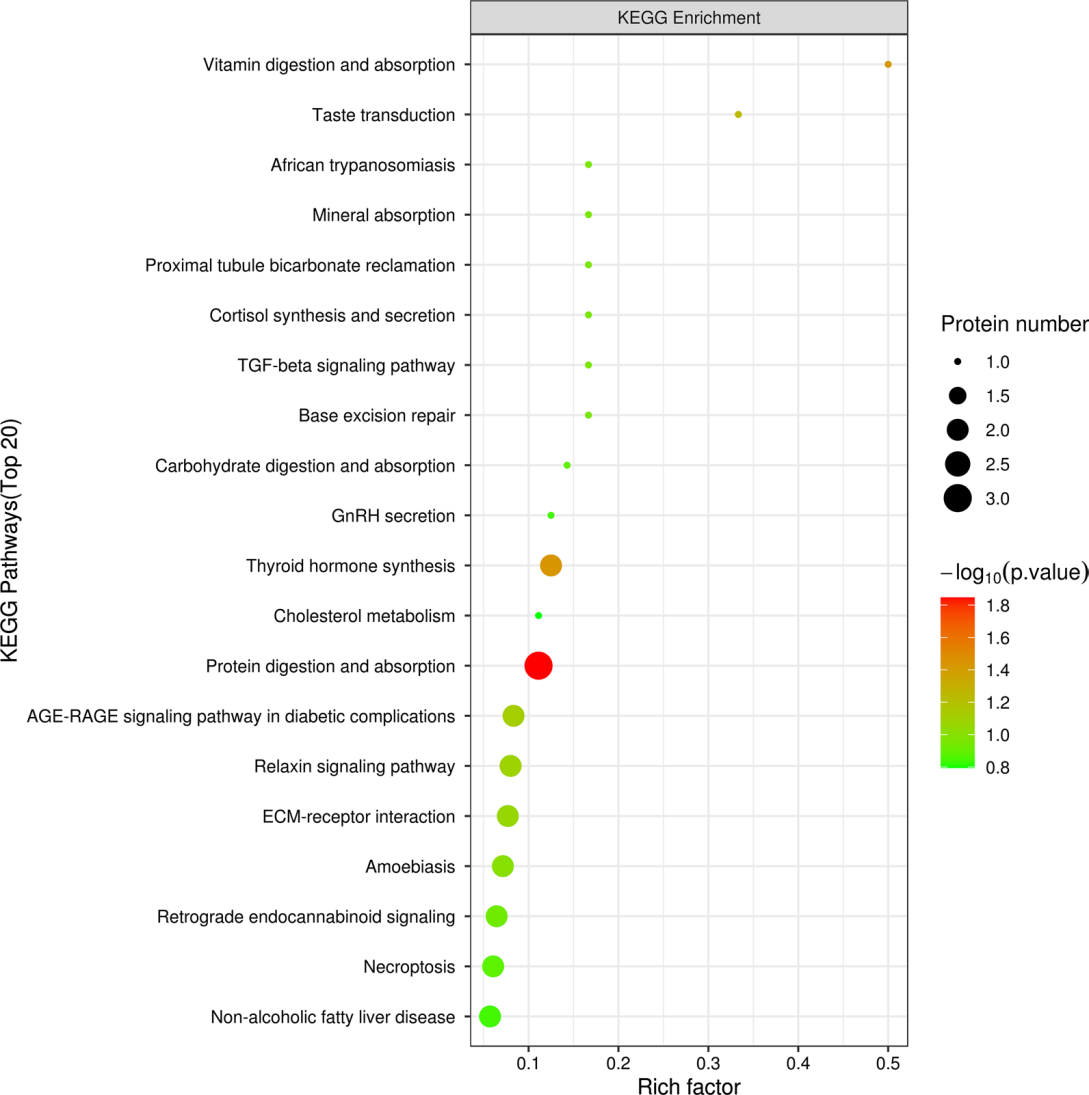
Bubble diagram of the KEGG pathway (top20) enrichment analysis of differentially expressed proteins

To analyze the biological process and drug action mechanism more systematically and comprehensively, we performed KEGG pathway enrichment analysis of the 65 differential proteins and found that 93 signaling pathways were changed significantly. The main signaling pathways involved included the protein digestion and absorption signaling, vitamin digestion and absorption signaling, Thyroid hormone synthesis (TH signaling) and TGF-beta (TGF-β) signaling pathways. The protein digestion and absorption pathways were visualized and analyzed, and the top 20 signaling pathways are shown in Fig. 5.

The abscissa in the graph is the enrichment factor (rich factor ≤ 1), which represents the proportion of the number of differentially expressed proteins annotated to a certain go functional category over the number of all identified proteins annotated to that go functional category.The ordinate represents the differential protein statistical outcome under each go functional classification; Where bubble color indicates the significance of enriched go functional categories, i.e., the p value was calculated based on Fisher's exact test, color gradient represents the magnitude of the p value (taken—log 10), and closer color to red represents smaller p value, corresponding to higher significance level of enrichment of go functional categories.

The abscissa of the plot shows the enrichment factor (richness factor ≤ 1). The enrichment factor indicates the number of differentially expressed proteins annotated to a GO functional category as a proportion of the number of all identified proteins annotated to the GO functional category. The ordinate represents the differential protein statistics under each KEGG pathway, and the bubble color represents the significance of the enriched KEGG pathways. Colors closer to red represent smaller p values, corresponding to a higher level of significance for metabolic pathway enrichment. The size of the circles indicates the number of proteins represented.

### Protein–protein interaction (PPI) analysis

**Fig. 6:**
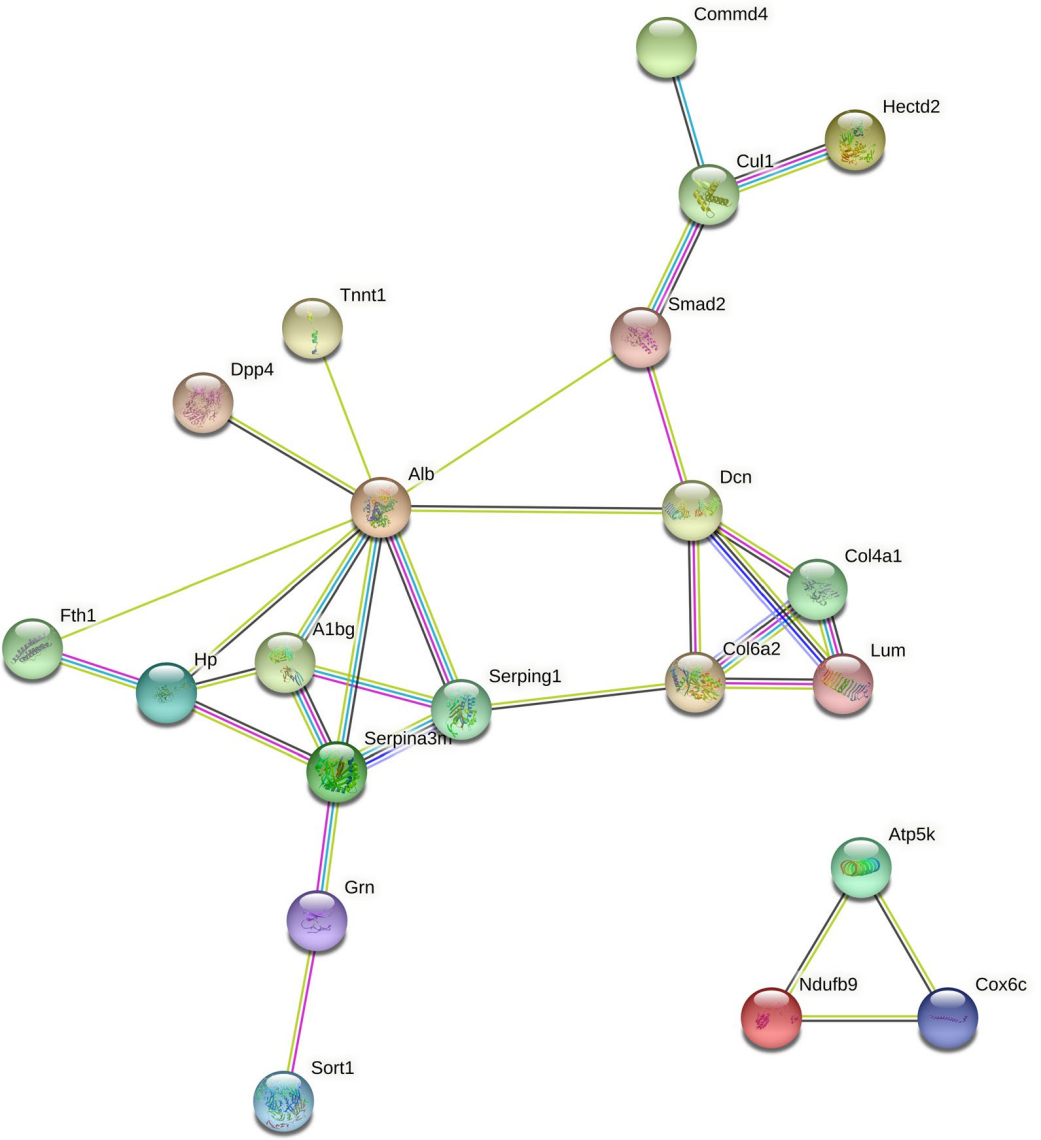
Interaction network analysis

An important mechanism by which proteins function is via interactions with other proteins and regulation of biological processes through protein-mediated pathways or the formation of complexes. According to PPI network analysis, a total of 21 among the 65 differentially expressed proteins may directly interact. Alb, DCN, Col6a2, and Smad2 were involved in many ways. Interestingly, we found that the differentially expressed protein DPP4 is also in figure, which is a protein closely related to diabetes. This protein may also be involved in and affect bone metabolic pathways. (see Fig. 6).

The nodes are proteins, the lines represent functional associations between the proteins, and the different line colors represent the types of evidence for the predicted functional associations.

## Discussion

OP is defined as a hip or lumbar BMD less than or equal to 2.5 standard deviations of the average BMD of the young reference population [31]. According to the guidelines of the International Osteoporosis Foundation [32], patients with osteopenia or OP should be treated with vitamin D combined with calcium [33], and patients with severe cases need supplementary drug intervention [2]. The use of calcium agents such as bisphosphate can improve the condition of patients with OP to some extent. However, these drugs have limited patient compliance and cause long-term adverse reactions [34], such as increased bone mineral density without improved bone quality or bone strength and effects on calcium and phosphorus metabolism [35, 36]. YGD treatment was shown to be effective, but the mechanism remains unclear. Quantitative mass spectrometry of proteins showed a possible relationship between pathways such as protein digestion and absorption signaling pathway obtained from GO enrichment analysis and bone metabolism, and protein network interaction showed that proteins such as albumin (Alb) have a relatively close relationship with OP. Thus, YGD may function through the process described above.

The normal digestion and absorption of proteins is fundamental for maintaining human life [38]. A variety of trace elements needed by the human body, such as vitamin B and cobalamin, must be absorbed through the intestine, and the absorption of cobalamin needs to be completed by binding proteins [39]. The protein digestion and absorption signaling pathway is mainly involved in the effective digestion and absorption of intestinal protein and fat [40]. Studies have shown that the effective digestion and absorption of proteins may be beneficial to cell metabolism, and high-quality cell status can help maintain immunity and functional stability in the body [41]. Dietary proteins participate in the physiological functions of the intestine to control amino acid, glucose and lipid metabolism so that bone cells can maintain normal physiological functions [42]. The protein digestion and absorption signaling pathway may play a role mainly through intestinal flora [43, 44], thus affecting bone calcium metabolism. By downregulating the key proteins in this signaling pathway, namely, Col6a2 and Col4a1, the protein DPP4 may be upregulated to promote bone formation and inhibit bone resorption. This confirmed the accuracy of our study that col6a2 and Col4a1 proteins were suppressed and that DPP4 protein was upregulated. There is a positive correlation between the vitamin digestion and absorption signaling pathways and aging [45]. Its relationship with protein digestion and absorption signal pathway is synergistic. With increasing age, the absorption of vitamins through the intestinal flora decreases significantly, the most important change is the decrease in vitamin D absorption, and this decrease in vitamin D absorption in turn leads to the occurrence of OP. In the vitamin digestion and absorption signaling pathway, we found that vitamin B was also involved [46]. The existing conclusions also confirm our findings that vitamins B1, B12 (cobalamin) and B9 (folic acid) have bone protective effects [47, 48]. Vitamin B12 deficiency can increase serum homocysteine levels, leading to OP and osteoporotic fractures [49]. In this study, we believe that the use of TCM may regulate the content of B vitamins in rats and protect bone tissue through this pathway. TH is directly involved in the development of osteocytes and controls the linear growth and maturation of bones [50], which requires protein to provide enough power. TGF-β is the main transforming growth factor, and the disturbance of TGF-β signaling is the basis of inflammatory diseases [51], which play an important role in OP [52–54]. This signaling pathway was also mentioned in our previous research [18]. Therefore, we believe that YGD may directly and indirectly participate in the regulation of this signaling pathway, thus affecting bone metabolism, and thereby controlling the occurrence and development of OP to a certain extent.

Interactive network analysis showed that Alb participates in a variety of processes, and related studies [55–57] have shown that the protein is closely related to energy and bone metabolism. Alb is abundant in human blood and is mainly involved in the transport of various substances. Alb also could enhance bone formation [58]. It has been reported that there is a direct relationship between serum Alb content and OP, and a low protein diet can lead to a decrease in bone density and eventually trigger OP [71]. According to our experimental findings, downregulation of Alb protein may be through the TH signaling pathway and thereby inhibit bone destruction [59, 69]. Through downregulating Alb thereby inhibited the expression of osteoclastogenic proteins and stimulated osteoclast apoptosis [72]. For preventing bone resorption. Decorin (DCN) is one of several glycosaminoglycan (GAG) no-collagen proteins secreted by bones. It is characterized as a leucine-rich repetitive core protein in bone [60]. DCN can directly interact with TGF-β in a dynamic environment to affect cell migration [61]. According to our study, the downregulation of DCN protein may be through TGF-β Signaling pathway inhibited the fibrotic response in skeletal muscle [70]. By decreasing the content of DCN, the acceleration of bone mineralization can be achieved [63]. Bone loss can be reduced by inhibiting the DCN protein. It can also promote the activity of osteoblasts [62]. Smad is the main transducer of bone morphogenetic protein (BMP) and the TGF-β signaling pathway that regulates mitochondrial function and apoptosis [64]. According to our results, the downregulation of Smad proteins similarly confirms that YGD may through TGF-β signaling pathway inhibits bone remodeling and reduces osteoclast activity [65]. Similarly, Smad inhibits the apoptosis of mature osteoblasts through BMP2 [66]. Interestingly, we found that DPP4 expression was significantly increased among the proteins regulated by YGD, and this protein was closely related to diabetes [67, 68]. The high expression of DPP4 in OP may indicate that different signaling pathways have beneficial effects on bone formation and bone resorption.

## Conclusions

Using TMT-based proteomics technology and proteome microarray technology, the effect of YGD on protein expression in bone tissue was studied. The results showed that YGD participated in several signaling pathways related to OP and some proteins involved in the regulation of signaling pathways. This confirmed the conclusions reached in some reports and proved our research hypothesis regarding YGD. That is, that YGD participates in the regulation of different target proteins, is involved in signal transduction via multiple signaling pathways and plays a therapeutic role. At the same time, we also found that there is a certain relationship between the differentially expressed protein DPP4 and OP, but how DPP4 participates in signal pathway transduction and affects the occurrence and development of OP is worthy of further study.

## Supplementary Information

**Additional file 1. **Identification versus quantification results statistical table.

**Additional file 2. **Table of differentially expressed protein statistics.

## Data Availability

The data are included in the article as figures and tables. All data generated during this study are included in this published article.

## Abbreviations

OP: Osteoporosis
YGD: Yigu decoction
TCM: Traditional Chinese medicine
TMT: Tandem mass tag
TGF-β: Transforming growth factor-beta
TRACP: Tartrate resistant acid phosphates
BGP: Bone Gla protein
DEGs: Differentially expressed genes
FA: Formic acid
FASP: Filter-aided sample preparation
BP: Biological process
CC: Cellular
MF: Molecular function
PPIs: Protein–protein interactions
TH: Thyroid hormone synthesis
ECM: Extracellular matrix
DCN: Decorin
GAG: Glycosaminoglycan
KEGG: Kyoto Encyclopedia of Genes and Genomes
KAAS: KEGG Automatic Annotation Server
BMD: Bone mineral density
Alb: Albumin
